# Supplementary material for: Analyses of Physical and Chemical Compositions of Different Medicinal Specifications of CRPV by Use of Multiple Instrumental Techniques Combined with Multivariate Statistical Analysis
Source: Molecules. 2022 May 20;27(10):3285. doi: 10.3390/molecules27103285 (PMC9148031; doi:10.3390/molecules27103285)
Supplement: Supplementary file 1 [file molecules-27-03285-s001.zip › molecules-1698243-supplementary.pdf]

molecules

Table S1. Information on Citri Reticulatae Pericarpium Viride.

Sample Number	Batch Number	Place of Origin	Sample Number	Batch Number	Place of Origin
G1	2007001	Jiangxi	S13	200501	Jiangxi
G2	200901	Hunan	S14	20200723	Jiangxi
G3	B810261-01	Hubei	S15	2101240082	Jiangxi
G4	1905002	Jiangxi	S16	190801	Jiangxi
G5	190701	Fujian	S17	200901	Jiangxi
G6	B2007151-01	Hubei	S18	20200914	Sichuan
G7	191201	Jiangxi	S19	190701	Sichuan
G8	2005130182	Fujian	S20	200701	Sichuan
G9	C340200601	Sichuan	S21	2101140285	Jiangxi
G10	200601	Fujian	S22	21011140276	Jiangxi
G11	2011010262	Fujian	——	——	——
G12	201211236	Jiangxi	——	——	——
